# Quality Parameters, Metabolomics Profile, and Biology Activities of Some Honey (*Apis mellifera*) Samples Obtained from Pretoria and Johannesburg, South Africa

**DOI:** 10.3390/metabo16100705

**Published:** 2026-09-23

**Authors:** Münire Turhan

**Affiliations:** Vocational School of Food, Agriculture and Livestock, Faculty of Agriculture, Bingöl University, 12000 Bingöl, Türkiye; mturhan@bingol.edu.tr

**Keywords:** South African honey, antioxidant activity, enzyme inhibition, chemical properties, antibiotic residues

## Abstract

**Background/Objectives:** Honey is a natural product with important nutritional and biological properties. This study aimed to comprehensively evaluate honey samples with regard to physicochemical quality, safety, antioxidant capacity, and enzyme inhibitory activities. **Methods:** Moisture, proline, diastase activity, electrical conductivity, free acidity, sugar composition, and hydroxymethylfurfural (HMF) were determined according to relevant Turkish Food Codex, Codex Alimentarius, and International Honey Commission criteria. Naphthalene and residues of 92 antibiotics were analyzed to assess potential contamination. Antioxidant activity and inhibitory effects against acetylcholinesterase (AChE), butyrylcholinesterase (BChE), and α-glycosidase were also evaluated. **Results:** The physicochemical parameters of the honey samples were generally within the established quality limits. Naphthalene was not detected in any sample, while none of the 92 analyzed antibiotics was detected above the limit of quantification. The samples exhibited measurable antioxidant activity and inhibitory effects against AChE, BChE, and α-glycosidase, indicating the presence of bioactive constituents that may contribute to these biological effects. **Conclusions:** The investigated honey samples demonstrated satisfactory physicochemical quality and safety, with no detectable naphthalene or antibiotic residues. Their antioxidant and enzyme inhibitory activities further highlight honey as a promising natural source of bioactive compounds with potential functional and health-promoting properties.

## 1. Introduction

Honey is a natural product with considerable nutritional and therapeutic potential, whose biological properties are largely attributed to its diverse composition of phenolic compounds, flavonoids, enzymes, organic acids, and other bioactive constituents. Honey has been reported to exhibit a broad range of biological activities, including antioxidant, antidiabetic, antimicrobial, anti-inflammatory, and anticholinesterase effects, which may contribute to its health-promoting potential. These properties can vary considerably depending on botanical and geographical origin, climatic conditions, soil characteristics, bee species, and post-harvest practices [1,2]. This variability affects not only the functional properties of honey but also its quality and authenticity. Inappropriate or uncontrolled use of antibiotics against bacterial diseases such as American and European foulbrood may result in residues in honey, posing risks to consumer health and international trade. Therefore, monitoring veterinary drug residues has become an important component of food safety, with multi-residue analyses commonly targeting sulfonamides, tetracyclines, macrolides, and quinolones [3]. Other bee products, including propolis, royal jelly, pollen, bee bread, apilarnil, and bee venom, also exhibit diverse biological activities, particularly antioxidant, antidiabetic, and anticholinesterase effects [4,5,6]. Among these products, honey is the most widely consumed.

Africa represents a geographically and botanically diverse region where honey production occurs under a wide range of ecological and climatic conditions. Previous studies have reported the presence of phenolic compounds, flavonoids, and other secondary metabolites in African honeys, which may contribute to their biological activities [7]. However, comprehensive studies simultaneously evaluating physicochemical quality parameters, safety indicators, and biological activities of African honeys remain limited. This gap highlights the need for integrated investigations to better characterize the quality, safety, and biofunctional properties of these honeys.

Key parameters used to assess honey quality include diastase activity, proline, moisture, electrical conductivity, sugar profile, free acidity, and insoluble solids. Diastase activity reflects honey freshness and possible heat treatment, whereas proline is a major amino acid associated with honey maturity, natural origin, and authenticity [2,8,9]. Moisture content is closely related to fermentation and microbial stability, while electrical conductivity provides an indirect indication of mineral, organic acid, and protein contents and is useful in honey classification [10]. The sugar profile, particularly fructose, glucose, and sucrose, is essential for evaluating nutritional characteristics and detecting adulteration [11].

Naphthalene analysis is relevant to food safety because it can reveal residues originating from chemicals used for moth control during honey storage [12]. Insoluble solids indicate mechanical purity and filtration efficiency, whereas free acidity reflects organic acid content and potential fermentation, thereby contributing to the assessment of honey quality and stability [13].

Phenolic acids and flavonoids are important secondary metabolites associated with the antioxidant capacity of honey. By scavenging free radicals, these compounds may help reduce oxidative stress and contribute to protection against chronic diseases [14,15,16,17,18,19,20,21]. Accordingly, DPPH, ABTS, and reducing-power assays are widely used to evaluate honey antioxidant activity.

Honey has also attracted increasing attention for its inhibitory effects on enzymes associated with neurodegenerative diseases. In particular, inhibition of acetylcholinesterase (AChE) and butyrylcholinesterase (BChE) is considered a relevant strategy in the management of Alzheimer’s disease [22,23,24]. Given their phenolic composition, African honeys may therefore represent promising natural sources of antioxidant and anticholinesterase compounds.

Additionally, the inhibition of carbohydrate digestion enzymes such as α-glycosidase and α-amylase is considered an effective approach to controlling postprandial hyperglycemia in the management of diabetes mellitus. The fact that naturally derived inhibitors have a lower side effect profile compared to synthetic agents highlights honey’s antidiabetic potential [25,26].

In this study, the quality parameters (diastase, proline, moisture, electrical conductivity, sugar profile, naphthalene, antibiotic residue, insoluble solids, and free acidity) of honey samples originating from South Africa were determined in detail; their secondary metabolite contents and antioxidant, anticholinergic, and antidiabetic activities were evaluated together. The study aims to provide a comprehensive contribution to the literature on the quality, reliability, and functional food potential of the analyzed honey samples.

## 2. Materials and Methods

### 2.1. Chemicals

All reagents and analytical standards required for LC-MS/MS, HS-GC-MS, and HPLC-RID-DAD analyses were obtained from Sigma-Aldrich (Steinheim, Germany). The chemicals and reference compounds used in the enzyme inhibition and antioxidant activity assays were also supplied by Sigma-Aldrich Chemie GmbH (Steinheim, Germany), and the enzymes employed in the experimental procedures were purchased from the same supplier. All reagents used throughout the study were of analytical grade purity.

### 2.2. Supply of Honey

Honey samples were obtained from the provinces of Pretoria (1. honey) and Johannesburg (2. honey) (South Africa) in May. The samples were classified as polyfloral honey based on information provided by the beekeepers regarding their botanical origin and production conditions. Following collection, all honey samples were collected using conventional methods without any processing and stored in airtight, light-protected containers at 4 °C until analysis to minimize potential changes in their physicochemical and bioactive properties.

### 2.3. Preparation of the Honey Extracts

A 5 g honey (*Apis mellifera*) sample was accurately weighed and extracted with 50 mL of a solvent mixture consisting of methanol and ultrapure water (50:50, *v*/*v*) containing 0.5% acetic acid. The mixture was agitated in an orbital shaker at 200 rpm for 30 min and subsequently subjected to ultrasonic treatment for 20 min to improve extraction efficiency. The samples were then centrifuged at 4000 rpm for 10 min to separate insoluble material. The clear supernatant was carefully collected and passed through a 0.22 μm PTFE syringe filter (25 mm diameter). The filtered extracts were transferred into 1.5 mL autosampler vials and injected into the analytical system for further analysis [2].

### 2.4. Secondary Metabolite Profiling by LC–HRMS

The LC–HRMS method used for the quantitative analysis of phenolic compounds was based on a previously developed and validated chromatographic method [27]. Briefly, the extracts were prepared at a concentration of 1 mg/mL, filtered through a 25 mm diameter PTFE syringe filter with a pore size of 0.22 μm (MARCEL SCHÜLLER GmbH, Berlin, Germany), and transferred into LC–MS vials prior to analysis.

The analyses were performed using an Ultra-High-Pressure Liquid Chromatograph (UHPLC, DIONEX UltiMate 3000 RS, Thermo Fisher Scientific, Waltham, MA, USA), equipped with a degasser, binary pump, autosampler, and column oven, coupled to an Exactive Plus Orbitrap high-resolution mass spectrometer (Thermo Fisher Scientific, Waltham, MA, USA) equipped with a heated electrospray ionization (HESI-II) interface. The UHPLC and HESI–Orbitrap HRMS systems were operated simultaneously using TraceFinder 3.2 software (Thermo Scientific), while data acquisition and recording were performed using Xcalibur software (version 2.1.0.1140, Thermo Fisher Scientific, Waltham, MA, USA).

Chromatographic separation was achieved using a reversed-phase Merck Purospher^®^ STAR RP-18 endcapped Hibar^®^ HR column (3 μm, 2.1 × 100 mm, Darmstadt, Germany). The column oven temperature was maintained at 30 °C. The mobile phases consisted of water containing 0.5% (*v*/*v*) glacial acetic acid (A) and LC–MS-grade methanol (B). The gradient program was as follows: 0% B (0–2 min), 0–98% B (2–15.5 min), 98% B (15.5–15.9 min), and 0% B (16–19 min). The flow rate was 300 μL/min, and the injection volume was 20 μL.

The Orbitrap mass spectrometer was operated in both positive and negative electrospray ionization modes. The ion source parameters were set as follows: spray voltage, 3.5 kV; sheath gas flow rate, 35 arbitrary units; auxiliary gas flow rate, 7 arbitrary units; sweep gas flow rate, 0; capillary temperature, 350 °C; auxiliary gas heater temperature, 350 °C; and S-lens RF level, 50. Data were acquired using Full MS and All Ion Fragmentation (AIF) modes. Full MS measurements were performed in both ESI+ and ESI− modes with the HCD cell closed, whereas AIF measurements were performed in both ionization modes with the HCD cell activated at a collision energy of 25 eV. The mass range was set to *m*/*z* 100–800, the resolving power was 17,500, the automatic gain control (AGC) target was 3 × 10^6^, and the maximum injection time was 2 ms. The mass accuracy was maintained at <5 ppm throughout the measurements. The Orbitrap mass spectrometer was calibrated in both positive and negative ionization modes using the corresponding Pierce™ LTQ Velos ESI calibration solutions according to the manufacturer’s instructions.

For quantitative determination of the selected phenolic compounds, calibration standards were prepared at concentrations of 10, 20, 40, 60, 80, 100, 200, 400, 600, 800, and 1000 ppb, and each calibration level was injected in triplicate. Quantification was performed using the corresponding calibration curves generated from the analytical standards.

#### LC–HRMS Compound Identification and Annotation

The identification and annotation of phenolic compounds were based on the combined evaluation of accurate mass measurements obtained from Full MS data and the corresponding fragmentation information obtained from AIF measurements. A mass accuracy threshold of <5 ppm was applied for precursor ion matching. The proposed molecular formulas were evaluated based on accurate mass, isotope patterns, and the observed adduct/ion forms in positive and negative ionization modes. Where applicable, the observed AIF fragment ions were compared with characteristic fragmentation patterns reported in the literature and with reference database information. Compounds for which authentic analytical standards were available were further confirmed by comparison of their retention times and mass spectral characteristics with those of the corresponding standards analyzed under identical chromatographic conditions.

Accordingly, compounds confirmed using authentic standards were considered identified compounds, whereas compounds assigned primarily on the basis of accurate mass, molecular formula, isotope/adduct information, and characteristic MS/MS fragmentation patterns were reported as putatively identified or annotated compounds. The metabolite annotation was therefore not based solely on exact-mass matching, but on the combined evidence obtained from accurate mass, ionization behavior, fragmentation characteristics, and reference information.

### 2.5. Total Phenolic and Flavonoid Content

The total phenolic content of bee products was determined using the Folin–Ciocalteu reagent (FCR) method [28], with gallic acid employed as the reference standard. For calibration, a stock solution of gallic acid (10 mg/mL) was prepared by dissolving 10 mg of gallic acid in 10 mL of distilled water. Aliquots ranging from 100 to 1000 μL of this stock solution were transferred into test tubes, and the volume was adjusted to 23 mL with distilled water. Subsequently, 0.5 mL of Folin–Ciocalteu reagent was added, and after 3 min, 1.5 mL of 2% (*w*/*v*) Na_2_CO_3_ solution was introduced. The mixtures were then allowed to react under ambient conditions for 2 h. Absorbance values were measured at 760 nm against a blank containing distilled water. Distilled water was used as a control in place of the sample, and the results were expressed as micrograms of gallic acid equivalents (µg GAE) [29]. The calibration curve of gallic acid is presented in Figure 1.

The total flavonoid content of bee products was determined according to the method described by Park et al. [30], using quercetin as the reference standard. For calibration, aliquots corresponding to 10, 20, 30, 40, and 50 μg of quercetin were prepared from a stock solution and transferred into test tubes. Subsequently, 0.1 mL of 1.0 M CH_3_COOK solution and 0.1 mL of 10% Al(NO_3_)_3_ solution were added. The reaction mixture was then brought to volume with 4.3 mL of ethanol and thoroughly mixed. After incubation at room temperature for 40 min, the absorbance was measured at 415 nm against a blank containing ethanol. The total flavonoid content of the samples was calculated from the calibration curve and expressed as micrograms of quercetin equivalents (µg QE). The calibration curve of quercetin is presented in Figure 2.

### 2.6. Antioxidant Activity Assay

Stock solutions of α-tocopherol, Trolox, BHT, and BHA used in all antioxidant activity assays were prepared in ethanol at a concentration of 7 mg/mL.

#### 2.6.1. Fe^3+^ Reduction Assay

The reducing power assay was performed according to the method described by Oyaizu (1986) with minor modifications [31,32,33]. Aliquots of the samples at the desired concentrations (15–60 µg/mL) were transferred into test tubes and mixed thoroughly using a vortex mixer. After the reaction steps, 1 mL of distilled water was added to each tube, vortexed, and the mixtures were kept in the dark for 10 min. Absorbance values were then measured at 700 nm, and all measurements were carried out in triplicate. Distilled water served as the blank, while the control was prepared by replacing the sample with distilled water under the same experimental conditions.

#### 2.6.2. Cu^2+^ Reducing Antioxidant Power Assay

The assay was carried out according to the method of Apak et al. [34] with slight modifications. Briefly, the reaction mixture was prepared in test tubes by adding 0.25 mL of CuCl_2_ solution (0.01 M), 0.25 mL of ethanolic neocuproine solution (7.5 × 10^−3^ M), and 0.25 mL of ammonium acetate (CH_3_COONH_4_) buffer solution (1.0 M, pH-adjusted according to the original protocol). Subsequently, honey samples at different concentrations (15–60 µg/mL) were added to the mixture. The final volume was adjusted to 2.0 mL with distilled water, and the reaction mixture was thoroughly mixed using a vortex to ensure complete homogenization. The tubes were then incubated at room temperature for 30 min under dark conditions to prevent light-induced degradation or interference. After incubation, the absorbance was measured spectrophotometrically at 450 nm against a reagent blank prepared in the same manner but without honey sample. All assays were conducted in triplicate, and results were expressed as mean values. An increase in absorbance corresponds to the formation of the Cu(I)-neocuproine complex, indicating higher cupric ion reducing capacity and thus stronger antioxidant potential of the honey samples.

#### 2.6.3. Ferric Reducing Antioxidant Power (FRAP) Assay

The ferric reducing antioxidant power (FRAP) assay was performed according to the method described by Bursal et al. with minor modifications [35]. The FRAP reagent was prepared by mixing TPTZ solution (10 mM, 2.25 mL) and FeCl_3_ solution (20 mM, 2.25 mL) in acetate buffer (0.3 M, pH 3.6) to obtain the working reagent. Bee product samples were prepared at different concentrations (15–60 μg/mL) in buffer, and 2.5 mL of each sample solution was mixed with 2.5 mL of freshly prepared FRAP reagent. The mixtures were incubated at 37 °C for 30 min in the dark, after which the reduction of the Fe^3+^–TPTZ complex was monitored by measuring absorbance at 593 nm. All measurements were performed in triplicate at each concentration.

#### 2.6.4. DPPH^•^ Scavenging Assay

The DPPH radical scavenging activity was determined according to the method of Blois [36]. A 1 mM DPPH solution was used as the radical source. Before the assay, the absorbance of the radical control prepared in ethanol was adjusted to 1.00 ± 0.025. Sample solutions at various concentrations (15–60 µg/mL) were transferred into test tubes, mixed thoroughly by vortexing, and incubated at room temperature in the dark for 30 min. After incubation, absorbance values were recorded at 517 nm against a blank containing ethanol.

#### 2.6.5. ABTS^•+^ Scavenging Assay

The ABTS radical cation scavenging activity was determined according to the method of Re et al. [37] with minor modifications based on a previous study. Before the assay, the absorbance of the control solution, consisting of ABTS^•+^ solution and phosphate buffer, was adjusted to 1.00 ± 0.025. Sample solutions at different concentrations (15–60 µg/mL) were mixed with 0.1 M phosphate buffer and 2 mM ABTS^•+^ solution in test tubes, vortexed, and incubated at room temperature in the dark for 30 min. Following incubation, absorbance values were measured at 734 nm. Phosphate buffer was used as the blank.

### 2.7. Enzyme Inhibition Assay

#### 2.7.1. Acetylcholinesterase (AChE) and Butyrylcholinesterase (BChE) Inhibition Assay

The inhibitory activities of the samples against acetylcholinesterase (AChE) and butyrylcholinesterase (BChE) were determined spectrophotometrically according to the method of Ellman et al. [22] with minor modifications [38]. In this assay, acetylthiocholine iodide and butyrylthiocholine chloride were used as substrates for AChE and BChE, respectively, while 5,5′-dithiobis-(2-nitrobenzoic acid) (DTNB) was employed as the chromogenic reagent. Briefly, sample solutions at various concentrations were mixed with phosphate buffer (0.1 M, pH 8.0) and the enzyme solution, and the mixture was pre-incubated for a short period at room temperature. After incubation, DTNB and the appropriate substrate were added to initiate the reaction. The formation of the yellow-colored 5-thio-2-nitrobenzoate anion was monitored by measuring absorbance at 412 nm using a spectrophotometer. All measurements were carried out in triplicate, and enzyme inhibition was calculated by comparing the reaction rates of the samples with those of the control containing no inhibitor.

#### 2.7.2. α-Glycosidase Inhibition Assay

The α-glycosidase inhibitory activity of the samples was determined according to a modified version of the method described by Tao et al. [25]. Briefly, sample solutions at different concentrations were mixed with α-glycosidase enzyme solution prepared in phosphate buffer (0.1 M, pH 6.8) and pre-incubated at 37 °C for 10 min. Subsequently, p-nitrophenyl-α-D-glucopyranoside (pNPG) was added as the substrate to initiate the reaction, and the mixture was further incubated at 37 °C. The reaction was terminated by the addition of sodium carbonate solution, and the release of p-nitrophenol was monitored by measuring absorbance at 405 nm using a spectrophotometer. A control containing all reagents except the sample was prepared under the same conditions, and all measurements were performed in triplicate. The percentage inhibition of α-glycosidase activity was calculated by comparing the absorbance values of the samples with those of the control.

### 2.8. Physicochemical Quality Parameter Assay

#### 2.8.1. LC–MS/MS Analysis of Veterinary Drug (Antibiotic) Residues

Preparation of veterinary drug solutions: Veterinary drug standards were prepared as stock solutions at a concentration of 1 mg/mL (1000 mg/kg). The resulting stock solutions were stored in the dark at temperatures below −20 °C. When preparing the working solution, a 1 mg/kg standard mixture working solution was prepared by diluting the 1000 mg/kg main stock solutions with a mixture of 50% (0.3% formic acid containing ultrapure water) and 50% (0.3% formic acid containing methanol). All solutions were stored in a deep freezer at −20 °C.

LC-MS/MS Instrument and Chromatographic Conditions for Veterinary Drug Analysis: The Shimadzu brand LCMS-8060NX model Liquid Chromatography-Triple Quadrupole Mass Spectrometer (LC-MS/MS) (Shimadzu Corporation, Kyoto, Japan) was used in veterinary drug residue analysis, with the Shimadzu DGU-40S Degassing Unit model degasser, the Shimadzu LC-40D XS Solvent Delivery Module model gradient pump, the Shimadzu SIL-40C XS model autosampler, the Shimadzu CTO-40S model column oven, and the Shimadzu LC MS-8060NX model modules. A Merck Supelco^®^ Purosper^®^ STAR RP-18 endcapped Hibar^®^ HR model UHPLC column (100 mm × 2.1 mm, 2 μm) was used for veterinary drug analysis. The column oven was set to 40 °C during analysis. An ultrapure water mixture containing 0.3% LC-MS grade formic acid was used for mobile phase A, and an LC-MS grade methanol mixture containing 0.3% LC-MS grade formic acid was used for mobile phase B. The mobile phase gradient was 1–5% B (0–1 min), 5–25% B (1–3 min), 25–30% B (3–4 min), 30–98% B (4–11 min), 98% B (11–13 min), and 98–1% B (13.1–18 min). The mobile phase flow rate was 0.3 mL/min, and the injection volume was 5 μL. A triple quadrupole mass spectrometer was used with an electrospray ionization (ESI) source capable of operating in both negative and positive ion modes [27].

#### 2.8.2. Naphthalene Analysis Assay

Naphthalene was determined according to the method described by İzol [12]. Briefly, 1 g of honey was weighed into a 20 mL headspace GC–MS vial. A naphthalene-D8 internal standard solution (10 mg/L in methanol) was added, and the vial was placed in the headspace autosampler for analysis. Measurements were performed using a PerkinElmer system consisting of a Clarus 690 gas chromatograph, a Clarus SQ 8T mass spectrometer, and a Turbomatrix 40 Trap headspace sampler. Calibration showed good linearity, with a correlation coefficient (R^2^) of 0.9959, and the limit of detection (LOD) was determined to be 0.5 µg/kg. Separation was carried out on an Elite-WAX capillary column (30 m × 0.25 mm i.d., 0.25 µm film thickness). The total analysis time was 10 min, with a solvent delay of 3 min; MS data acquisition started at 3.5 min and continued for 10 min over a mass range of m/z 35–250. Data processing was performed using Turbomass software (version 6.1.2), and compound identification was confirmed by comparison with spectra from the NIST library integrated into the instrument software.

#### 2.8.3. Sugar Composition Analysis by HPLC-RID

The sugar profile of the honey samples was determined by high-performance liquid chromatography (HPLC) equipped with a refractive index detector (RID), following the procedures described by the Turkish Standards Institute (TS 13359) [27] and the International Honey Commission (IHC) [2]. For sample preparation, 5 g of honey was accurately weighed and dissolved in approximately 40 mL of ultrapure distilled water. Subsequently, 25 mL of methanol was added, and the mixture was transferred to a 100 mL volumetric flask and diluted to volume with ultrapure distilled water. The resulting solution was filtered through a disposable 0.45 μm syringe filter, and about 1.5 mL of the filtrate was transferred into HPLC vials for analysis. Sugar identification and quantification were performed using calibration curves obtained from standard sugar solutions, and the results were expressed as percentages. HPLC analyses were carried out under the following chromatographic conditions: injection volume of 10 μL, column/detector temperature maintained at 30 °C, flow rate of 1.3 mL/min, and a mobile phase consisting of ultrapure water and acetonitrile (20:80, *v*/*v*).

#### 2.8.4. Hydroxymethylfurfural (HMF) Analysis by HPLC-DAD

The hydroxymethylfurfural (HMF) content of honey samples was determined by high-performance liquid chromatography (HPLC) according to the method described by Bogdanov et al. [39], in accordance with the International Honey Commission (IHC) and Turkish Standards Institute (TS 13356) [27] procedures. For sample preparation, 5 g of honey was accurately weighed into a 50 mL volumetric flask and dissolved in 25 mL of ultrapure distilled water with thorough mixing. Subsequently, 0.5 mL of Carrez I solution was added and mixed, followed by the addition of 0.5 mL of Carrez II solution, and the mixture was homogenized. The volume was then adjusted to 50 mL with ultrapure distilled water and allowed to stand for 3 h to ensure complete clarification. After incubation, the solution was filtered, and an aliquot of the filtrate (approximately 1.5 mL) was further filtered through a 0.22 μm membrane filter, transferred into HPLC vials, and injected into the HPLC system. Quantification of HMF was carried out by comparing the peak areas of the samples with those obtained from standard HMF solutions. Chromatographic separation was performed using a C18 column (4.6 × 250 mm) with a diode array detector (DAD). The column temperature was maintained at 30 °C, the flow rate was set to 1.0 mL/min, and the injection volume was 20 μL. The mobile phase consisted of methanol and ultrapure distilled water (10:90, *v*/*v*).

#### 2.8.5. Proline Analysis

The proline content of honey samples was determined according to the method described by İzol et al. [2], in accordance with the procedures recommended by the International Honey Commission (IHC) and the Turkish Standards Institute (TS 13357) [27]. All reagents and solutions were prepared as specified in the reference method.

Briefly, 5 g of honey was accurately weighed into a 100 mL volumetric flask, dissolved in 50 mL of distilled water, and diluted to volume with distilled water. An aliquot of 0.5 mL of this solution was transferred into a test tube. For comparison, 0.5 mL of distilled water was used as a blank, and 0.5 mL of proline standard solution was placed in a separate tube. To each tube, 1 mL of ninhydrin solution and 1 mL of formic acid were added, and the mixtures were thoroughly mixed and allowed to stand for 15 min. The tubes were then heated in a boiling water bath for 15 min, followed by incubation in a second water bath at 70 °C for 10 min. After cooling, 5 mL of 50% (*v*/*v*) 2-propanol was added to each tube, and the mixtures were kept at room temperature for 45 min to allow color development. Absorbance was measured at 510 nm using a UV–Vis spectrophotometer. Proline concentrations were calculated according to the equation provided in the reference method.

#### 2.8.6. Diastase Analysis

Diastase activity was determined according to the method described by İzol et al. [2], consistent with the procedure recommended by the International Honey Commission (IHC). Briefly, 1 g of honey was accurately weighed and diluted to volume with acetate buffer in a 100 mL volumetric flask. An aliquot of 5 mL of this solution was transferred into a test tube, while 5 mL of acetate buffer was placed in a separate tube to serve as the blank. Both tubes were pre-incubated in a water bath at 40 °C for 5 min, after which one Phadebas tablet was added to each tube. The mixtures were vortexed for approximately 10 s and returned to the water bath for incubation. After 30 min, 1 mL of NaOH solution was added to stop the reaction, and the tubes were mixed thoroughly. The samples were then centrifuged at 3600 rpm for 5 min, and the absorbance of the supernatant was measured at 620 nm using a UV–Vis spectrophotometer, with distilled water as the reference.

#### 2.8.7. Moisture Content Analysis

The moisture content of honey samples was determined in accordance with the methods recommended by the International Honey Commission (IHC) and the Turkish Standards Institute (TS 13365) [27]. Measurements were performed using a digital honey refractometer calibrated with distilled water at 20 °C prior to analysis. A small, homogenized portion of honey was carefully placed on the prism surface to ensure complete coverage without air bubbles, and the refractive index was recorded after temperature equilibration. Moisture content was calculated from the measured refractive index values using standard honey moisture conversion tables. Each sample was analyzed in triplicate, and the results were expressed as percentage moisture (%, *w*/*w*) [2].

#### 2.8.8. Electrical Conductivity Analysis

The electrical conductivity of honey samples was determined in accordance with the method recommended by the International Honey Commission (IHC) and the Turkish Standards Institute (TS 13366) [27]. Briefly, honey samples were dissolved in distilled water to obtain a solution corresponding to 20% (*w*/*v*) dry matter. The electrical conductivity of the solution was measured at 20 °C using a calibrated conductivity meter. Prior to measurements, the instrument was calibrated with standard potassium chloride (KCl) solutions of known conductivity. All measurements were performed in triplicate, and the results were expressed as mS/cm at 20 °C [2].

#### 2.8.9. Free Acidity Analysis

The free acidity of honey samples was determined according to the method specified in the Turkish Standards Institute (TS 13360) [2,27]. Briefly, 10 g of honey was weighed into a 250 mL beaker and dissolved in 75 mL of ultrapure distilled water with continuous stirring. The resulting solution was titrated with 0.05 N NaOH using phenolphthalein as an indicator until the endpoint at pH 8.3 was reached, indicated by the appearance of a persistent pale pink color. The free acidity of the honey samples was calculated using the formula provided in the reference method and expressed as milliequivalents of acid per kilogram of honey (meq/kg).

#### 2.8.10. pH Analysis

The pH of honey samples was measured using a calibrated digital pH meter. Briefly, 10 g of honey was weighed into a 100 mL beaker and dissolved in 75 mL of ultrapure distilled water with gentle stirring. Prior to measurement, the pH meter was calibrated using standard buffer solutions at pH 4.00, 7.00, and 10.00. The pH value was then recorded by immersing the electrode directly into the prepared honey solution, and measurements were performed in triplicate [2].

### 2.9. Statistical Analysis

All antioxidant activity measurements were performed in triplicate as technical replicates for each independent honey sample, and the results were expressed as mean ± standard deviation (SD). The triplicate measurements were used to assess analytical repeatability and were not considered independent biological replicates. Accordingly, the results were evaluated descriptively based on the mean values and standard deviations obtained from the technical replicates. Statistical analyses and data processing were performed using IBM SPSS Statistics, version 27.0 (IBM Corp., Armonk, NY, USA) software, while Microsoft Excel was used for preliminary data processing and graphical presentation. Given the limited number of independent honey samples, the findings were interpreted cautiously and are considered descriptive of the investigated samples rather than statistically generalizable to the wider honey population.

## 3. Results

### 3.1. Metabolomics Profile by LC–HRMS

In this study, the phenolic compound profiles of two different honey samples were determined in detail using the LC-MS/MS method, and it was found that both samples had a composition rich in phenolic acids and flavonoids. The secondary metabolite profile results of the honey samples are presented in Table 1. The phenolic profile of honey can show significant differences depending on plant origin, geographical conditions, and environmental factors, and these differences directly affect the biological activities of honey [27,40].

The following compounds were not detected in either of the two honey samples: 3-hydroxybenzoic acid (3-HBA), 3-hydroxyphenylacetic acid (3-HPA), gallic acid (3,4,5-trihydroxybenzoic acid), Protocatechuic acid (3,4-Dihydroxybenzoic acid), Protocatechuic acid ethyl ester (Ethyl 3,4-Dihydroxybenzoate), 3,4-dihydroxybenzaldehyde (Protocatechuic aldehyde), 2,4-dihydroxybenzoic acid (beta-Resorcylic acid), Homovanillic acid (4-Hydroxy-3-methoxyphenylacetic acid), Vanillin, Gentisic acid, 3,4-Dihydroxyphenylacetic acid (DOPAC, Homoprotocatechuic acid), trans Cinnamic acid, 3-(4-Hydroxyphenyl) propionic acid, α-Cyano-4-hydroxycinnamic acid, Catechin (Cianidanol)-p, Epigallocatechin, Vicenin, 2 Apigenin 7-glucuronide, Apigenin 7-glucoside, Apiin (Apigenin-7-(2-O-apiosylglucoside) Vitexin (Apigenin 8-C-glucoside), Schaftoside, Ethylgallate, Luteolin-7-O-glucuronide (Luteolin-7-O-β-D-glucuronide), Lute-oloside (Luteolin 7-glucoside), Luteolin 7-rutinoside, Narcissin (Narcissoside, Isorham-netin 3-rutinoside), Orientin, Kaempferitrin, Nicotiflorin (Kaempferol 3-rutinoside, Kaempferol 3-O-β–rutinoside), Tiliroside, Leucoside (Kaempferol 3-sambubioside), Fisetin hydrate, Naringin, Sakuranetin (Naringenin 7-O-methyl ether), Narirutin (Nariru-tinsa, Naringenin rutinoside), Oleuropein, Eriodictyol, Liquiritigenin, Liquiritin (4′,7-Dihydroxyflavanone 4′-glucoside), Genistein (5,7-Dihydroxy-3-(4-hydroxyphenyl)-4H-chromen-4-one), Daidzin, Formononetin (Neo-chanin), Doxorubicin Hydrchloride, Esculin hydrate, Phloridzin, Rosmarinic acid, Glabridin, Arbutin, Emodin, Etoposide, and Hesperidin [27].

### 3.2. Antioxidant Activity Results

The antioxidant activities of the honey samples were evaluated using the DPPH^•^ and ABTS^•+^ radical scavenging methods, and the results were expressed as IC_50_ values (Table 2). The findings obtained showed that both honey samples possessed free radical scavenging capacity.

In the DPPH test, the IC_50_ value of honey sample 1 was determined to be 41.443 ± 2.185 μg/mL, while the IC_50_ value of honey sample 2 was determined to be 32.621 ± 2.045 μg/mL. In the ABTS test, the values obtained were 45.110 ± 3.108 μg/mL and 30.335 ± 2.795 μg/mL, respectively. These results reveal that the second honey sample, in particular, has a higher antioxidant capacity against both radical systems. Compared to standard antioxidants, the activity of the honey samples was found to be close to that of synthetic antioxidants. In particular, the IC_50_ values of the second honey sample in the DPPH and ABTS tests were in a similar range to those of BHT and Trolox, indicating that honey has significant potential as a natural antioxidant source. Studies on honey from different geographical and botanical origins show that antioxidant activity can vary widely. These differences are mainly attributed to the variability in phenolic content and compound profile [41]. This also explains the different IC_50_ values observed between the two honey samples in our study. Indeed, the detection of higher levels of flavonoids such as luteolin, kaempferol, quercetin, and isorhamnetin in the second honey sample may have contributed to its stronger antioxidant activity. The antioxidant capacity of honey largely stems from its phenolic acids and flavonoids. The literature reports that honey’s antioxidant activity is due to the synergistic effect of many compounds, such as phenolic compounds, flavonoids, ascorbic acid, amino acids, and Maillard reaction products [40].

The Fe^3+^ reducing, Cu^2+^ reducing, and Fe^3+^–TPTZ (FRAP) methods used to determine antioxidant capacity are common analyses based on reduction power that reveal the electron-donating ability of samples. The results are shown in Table 3.

According to the results obtained, both honey samples showed a certain level of reduction capacity in all test systems. However, it was observed that the reduction power of honey samples was lower compared to standard antioxidants such as BHA, BHT, α-tocopherol, and Trolox. This is an expected result, as standard antioxidants are pure compounds, while honey is a complex natural matrix containing antioxidant components at lower concentrations. Among the standards, α-tocopherol was found to have a very high Cu^2+^ reduction capacity, while BHA exhibited strong activity in Fe^3+^ reduction and FRAP methods. These findings are consistent with studies reporting that strong lipophilic and phenolic antioxidants can exhibit high activity in electron transfer-based systems.

When honey samples were compared, honey sample No. 2 showed higher activity than honey sample No. 1 in both Fe^3+^ and Cu^2+^ reduction tests. This difference may be due to factors such as the botanical origin of the honey, its phenolic and flavonoid content, mineral composition, harvest period, and storage conditions. The literature reports that dark-colored honeys with high phenolic content generally exhibit stronger reduction capacity [42,43]. When examining the FRAP method results, it is seen that both honey samples have the ability to reduce ferric ions, but these values remain lower than the standards.

The total phenolic and flavonoid results for the analyzed honey samples are presented in Table 4. When examining the total phenolic and total flavonoid contents of the honey samples, it was determined that both samples contained measurable levels of phenolic and flavonoid compounds.

The total phenolic compound content was found to be 34,121 ± 1028 µg GAE/g in honey sample 1 and 57,233 ± 2022 µg GAE/g in honey sample 2. Similarly, the total flavonoid content was determined to be 27,854 ± 0.983 µg QE/g in honey sample No. 1 and 33,212 ± 0.995 µg QE/g in honey sample No. 2. These results show that honey sample No. 2 is richer in both phenolic compounds and flavonoids. It has been reported that the total phenolic content of honeys from different geographical and botanical origins varies widely.

### 3.3. Anticholinergic and Antidiabetic Properties Results

The anticholinergic effect was determined by AChE and BChE enzyme inhibition, while the antidiabetic effect was assessed via α-glycosidase enzyme inhibition. The inhibitory effects of honey samples on AChE, BChE, and α-glycosidase enzymes were evaluated based on IC_50_ values. The results of AChE, BChE, and α-glycosidase enzyme inhibition by honey samples are presented in Table 5.

According to the results obtained, both honey samples were found to have an inhibitory effect on three enzymes. In terms of AChE inhibition, honey sample 2 (IC_50_: 8.21 µg/mL) showed stronger activity compared to honey sample 1 (IC_50_: 11.33 µg/mL). In terms of BChE inhibition, the activity of both samples was found to be similar (IC_50_: 13.11 and 13.63 µg/mL). In terms of α-glycosidase inhibition, honey sample 2 (IC_50_: 9.33 µg/mL) was found to exhibit a higher inhibitory effect than honey sample 1 (IC_50_: 10.71 µg/mL). When compared to reference inhibitors, pure inhibitors such as tacrine and acarbose were found to have lower IC_50_ values; however, it can be stated that the honey samples exhibited a remarkable level of inhibitory activity for a natural product.

### 3.4. Physicochemical Quality Parameter Results of Honey Samples

The physicochemical quality parameters of the honey samples, including naphthalene content, sugar composition (glucose, fructose, sucrose, maltose, fructose + glucose, and fructose/glucose ratio), proline content, diastase activity, HMF level, moisture, electrical conductivity, free acidity, and pH values, are presented in Table 6. These parameters are widely used to evaluate honey quality, freshness, botanical origin, and possible exposure to heat or storage conditions. In particular, HMF and diastase activity are considered important indicators of thermal processing and aging, while moisture and free acidity are closely related to fermentation risk and storage stability. In addition, proline content and sugar profile provide valuable information regarding the maturity of honey and its authenticity. Therefore, the evaluation of these parameters is essential for assessing both the nutritional quality and the compliance of honey samples with international honey standards.

Proline content is an important indicator of honey’s maturity and authenticity. In this study, 521.854 mg/kg of proline was detected in honey sample 1 and 759.95 mg/kg in honey sample 2. According to the Turkish Food Codex Honey Regulation, the proline content must be at least 300 mg/kg. According to the International Honey Commission and Codex Alimentarius criteria, a proline content generally higher than 300 mg/kg in flower honeys indicates that the honey is mature and natural. The fact that the obtained values are well above this limit indicates that the samples are mature and unadulterated honey. The literature reports that proline values in honey obtained from different regions generally range from 300 to 800 mg/kg [2,8,40,44]. In this respect, the results obtained are consistent with the literature.

The antibiotic results for the honey are presented in Table 7. In both honey samples, all 92 different antibiotics analyzed quantitatively were below the limit of quantification (LOQ) and were not detected. These results indicate that the honey samples examined are safe in terms of veterinary drug residues.

Antibiotic residues in honey are an important food safety concern, as veterinary drugs used in apiculture may persist in the final product. Historically, antibiotics such as tetracyclines, sulfonamides, aminoglycosides, and macrolides were widely used to control bacterial diseases like American foulbrood, and these compounds are among the most frequently reported residues in honey [45,46].

## 4. Discussion

The phenolic and flavonoid profiles of the two honey samples revealed substantial qualitative and quantitative differences, highlighting their distinct phytochemical compositions and, consequently, their potential biological activities. Both samples contained a broad spectrum of bioactive compounds, including phenolic acids, flavonoids, flavonols, flavones, and flavanones, which are well recognized for their antioxidant, anti-inflammatory, antimicrobial, cardioprotective, and anticancer properties. The analysis revealed that the honey 1 and honey 2 samples exhibited a rich secondary metabolite profile. Both honey samples contained phenolic acids such as 4-Hydroxybenzoic acid, Salicylic acid, Syringic acid, Vanillic acid, *p*-Coumaric acid, Caffeic acid, and Ferulic acid, as well as flavonoids such as Chrysin, Apigenin, Luteolin, Quercetin, and Pinocembrin. Caffeic acid and ferulic acid, in particular, exhibit electron-donating properties due to the presence of phenolic hydroxyl groups and play an active role in neutralizing DPPH and ABTS radicals [47]. The presence of pinocembrin, naringin, and chrysin in particular suggests that these honeys may be typical propolis-enriched honeys or have specific flora such as forest rose/chestnut. Pinocembrin is a potent antioxidant commonly found in propolis and certain monofloral honeys; it also has antimicrobial, anti-inflammatory, and neuroprotective potential [48,49,50]. The chemical composition of honey is directly dependent on the flora visited by bees.

Among the identified compounds, kaempferol was the predominant flavonoid in honey 2 (12,123.28 µg/kg), representing the highest concentration detected in the entire dataset. Kaempferol is a potent antioxidant known for its remarkable free radical scavenging capacity, metal-chelating properties, and ability to modulate intracellular signaling pathways. Moreover, kaempferol has been widely investigated for its chemopreventive properties, including induction of apoptosis and inhibition of cancer cell proliferation [50,51]. The exceptionally high concentration observed in honey 2 strongly suggests a superior antioxidant and anti-inflammatory potential.

Luteolin was another major constituent, particularly abundant in honey 2 (2425.16 µg/kg) compared with honey 1 (66.34 µg/kg). Luteolin is considered one of the most bio-logically active flavones, with strong antioxidant, neuroprotective, anti-inflammatory, and anticancer potential. Its capacity to inhibit ROS generation, downregulate inflammatory mediators, and protect neuronal cells against oxidative damage has been extensively documented [42,52,53]. The high luteolin content likely contributes substantially to the elevated bioactivity of honey 2.

Similarly, apigenin showed a striking difference between samples, reaching 778.36 µg/kg in honey 2 versus only 25.67 µg/kg in honey 1. Apigenin has strong antioxidant, anxiolytic, anti-inflammatory, and anticancer potential. It is known to inhibit acetylcholinesterase and butyrylcholinesterase enzymes and to modulate multiple signaling pathways, including PI3K/Akt, MAPK, and NF-κB, thereby inhibiting tumor growth and inflammatory responses [54,55,56]. The relatively high apigenin concentration may be associated with the observed in vitro biological activity of the honey 2.

Among flavonols, quercetin and isorhamnetin were detected at appreciable levels in both samples, with notably higher concentrations in honey 2. Quercetin reached 778.33 µg/kg in honey 2, while isorhamnetin attained 689.79 µg/kg. Quercetin is widely regarded as one of the most effective natural antioxidants, demonstrating strong radical scavenging, anti-inflammatory, antiviral, and cardioprotective activities [57]. Isorhamnetin, a methylated derivative of quercetin, exhibits similar pharmacological potential, including enhanced bioavailability and anti-atherosclerotic and antitumor properties [58].

Neohesperidin was exclusively detected in honey 2 (805.99 µg/kg). This flavanone glycoside is known to have antioxidant, hepatoprotective, cholesterol-lowering, and antidiabetic potential. Several studies have highlighted its ability to regulate glucose metabolism and reduce oxidative stress in metabolic disorders [59]. Its presence may provide additional functional benefits unique to honey 2.

Also, myricetin, which is specific to honey 2 (949.34 µg/kg), is a highly potent antioxidant with demonstrated neuroprotective, antidiabetic, antimicrobial, and anticancer potential. Its ability to inhibit lipid peroxidation and modulate glucose homeostasis has attracted considerable attention [60]. The detection of myricetin further distinguishes honey 2 as a richer source of pharmacologically relevant flavonoids.

Pinocembrin, abundant in both samples and especially in honey 2 (586.12 µg/kg), is a characteristic flavanone frequently associated with propolis and certain unifloral honeys. Pinocembrin is considered a marker for honey freshness and quality. Pinocembrin has strong antimicrobial, neuroprotective, cardioprotective, and antioxidant potential. It has demonstrated efficacy against Gram-positive bacteria and has been investigated as a promising neuroprotective agent in ischemic injury models [61].

Chrysin and galangin, two flavonoids commonly associated with bee products, were detected in both honeys at higher concentrations in honey 2. While chrysin exhibits anti-inflammatory, anxiolytic, and anticancer potential, galangin is characterized by strong antimicrobial, antioxidant, and antitumor activities. Their simultaneous presence suggests a strong contribution from propolis-derived phytochemicals [62].

Phenolic acids also contributed significantly to the antioxidant potential. 4-Hydroxybenzoic acid was the dominant phenolic acid in both samples, particularly in honey 2 (2190.82 µg/kg). This compound exhibits notable antimicrobial and antioxidant properties. Coumaric acid, syringic acid, vanillic acid, caffeic acid, and ferulic acid were also abundant. Caffeic acid is particularly noteworthy due to its well-documented antioxidant, anti-inflammatory, and anticancer potential, largely attributed to its ability to inhibit oxidative stress and inflammatory signaling pathways [63].

Quinic acid was detected exclusively in honey 1 at a remarkably high concentration (1513.72 µg/kg). This organic acid has been associated with antioxidant, antiviral, and anti-inflammatory effects [43]. Likewise, naringenin was highly abundant only in honey 1 (763.76 µg/kg), suggesting that honey 1 may possess unique hepatoprotective and lipid-lowering properties, as naringenin has been shown to improve glucose metabolism and attenuate hepatic steatosis [54]. Furthermore, the presence of certain compounds, such as chlorogenic acid and quinic acid, only in the first honey sample suggests that the botanical origins of the samples may be different. Chlorogenic acid has been reported to exhibit an inhibitory effect on enzymes associated with carbohydrate metabolism and to have antidiabetic potential [64]. The high levels of myricetin (949.34) and ellagic acid (411.42) found in honey 2 indicate that this honey has a different botanical source (possibly heath- or citrus-dominated flora). The presence of ellagic acid, in particular, indicates the presence of plants belonging to the heather or heath family (Ericaceae) [48].

The exclusive occurrence of rutin, astragalin, ellagic acid, and CAPE in honey 2 further enriches its functional profile. CAPE is recognized as one of the most potent bioactive compounds in bee products, even at relatively low concentrations, and has the potential to exert remarkable anti-inflammatory, immunomodulatory, and anticancer potential by sup-pressing NF-κB-related signaling pathways [65].

In summary, honey 2 demonstrated markedly higher concentrations and a greater diversity of phenolic compounds than honey 1. This richer phytochemical profile may strongly account for its superior antioxidant, anti-inflammatory, antimicrobial, and chemopreventive potential. In contrast, honey 1 exhibited a more limited but distinct composition, characterized by high levels of quinic acid and naringenin, which may confer specific metabolic and hepatoprotective benefits. These findings are consistent with previous studies reporting that variations in floral origin, geographical location, and environmental conditions significantly influence honey phenolic composition and biological activity [42,43].

The substantially higher levels of kaempferol, luteolin, apigenin, quercetin, myricetin, and neohesperidin in honey 2 may be associated with the observed in vitro biological activities of the sample. However, the unique presence of quinic acid and naringenin in honey 1 suggests that both honeys may offer valuable yet distinct health-promoting properties. These compositional differences may explain potential variations in their antioxidant, enzyme inhibitory, and antimicrobial activities observed in subsequent analyses.

Alvarez-Suarez et al. [42] reported that caffeic and ferulic acids are among the most common phenolic acids in European and South American honeys. Similarly, Cianciosi et al. [43] reported that coumaric acid and vanillic acid are frequently detected compounds in honey samples from different floral sources. This situation shows that the results obtained are consistent with the literature. In a previous study, it was reported that honey samples with high luteolin and quercetin content had higher total antioxidant capacity [42]. The failure to detect certain phenolic compounds in the study can be explained not by their complete absence, but rather by their concentrations falling below the detection limit, their natural absence in the nectar source, or by the plant source not producing these metabolites. Similar situations have been reported in honey samples obtained from different geographical regions [2].

The findings indicate that the phenolic compound profile of honey may serve not only as a chemical quality indicator but may also be associated with its observed antioxidant and enzyme inhibitory activities. The number of hydroxyl groups, degree of conjugation, and glycosidic structures of phenolic compounds may influence their biological activity. Therefore, honey samples with higher levels of flavonoids may contribute to the observed in vitro bioactive potential of honey; however, further studies are required to establish the specific contribution of individual phenolic compounds to these activities. These results once again highlight the importance of honey as a functional food and a source of natural bioactive compounds.

Studies conducted with honey samples obtained from different regions have reported that DPPH IC_50_ values generally ranged between 20 and 80 μg/mL [42]. The fact that the values obtained in this study fall within this range indicates that the results are consistent with the literature. These data indicate that the antioxidant capacity of the second honey sample, in particular, is similar to or better than that of many honey samples reported in the literature.

The antioxidant capacity of honey is related not only to the amount of phenolic compounds but also to the diversity of compounds and synergistic interactions. Shahidi and Ambigaipalan [47] reported that the co-presence of phenolic compounds can increase total antioxidant activity, and this effect may be higher than the effect of individual compounds. In our study, the higher phenolic diversity in the second honey sample is consistent with lower IC_50_ values.

The FRAP method particularly reflects the effect of hydrophilic antioxidants more clearly. Therefore, it is thought that some antioxidant components contained in honey may play a more effective role under different pH conditions or with different reaction mechanisms. Similarly, previous studies have indicated that the antioxidant capacity of honey may vary depending on the method used and that different test systems reflect different antioxidant properties [66]. It has been reported that FRAP values in European and Mediterranean honeys correspond to moderate antioxidant capacity and that these values are directly related to the amount of phenolic compounds [67]. Similarly, recent studies on African and Middle Eastern honeys have emphasized that Cu^2+^ reduction capacity is related to the mineral content and flavonoid profile of honey [68]. Recent research has shown that evaluating honey’s antioxidant capacity using a single method is insufficient and that using tests based on different mechanisms together yields more reliable results [69].

Many studies have reported that these values can vary in the range of approximately 20–100 µg GAE/g, while flavonoid contents are generally lower but variable [69]. The results obtained in this study are consistent with those reported in the literature, and it can be said that the honey samples examined are moderately rich in phenolic compounds.

In the study conducted by Serem and Megan, honeys collected from different regions of South Africa were evaluated in terms of total phenolic content, flavonoid content, and antioxidant activity using the DPPH method. The total phenolic content was reported to range between 68.85 and 167.96 mg GAE/100 g, while the total flavonoid content varied from 14.66 to 51.6 mg CE/100 g. The DPPH antioxidant activity was determined to be in the range of 0.42–3.72 µmol TE/g [70]. In comparison, the antioxidant results obtained in the present study are consistent with those reported in the literature; however, the total phenolic and flavonoid contents were found to be comparatively lower. This discrepancy may be attributed to differences in the floral diversity of the geographical regions where the honeys were produced.

When the phenolic profile determined by LC-HRMS analysis was examined, phenolic acids and flavonoids such as 4-hydroxybenzoic acid, syringic acid, vanillic acid, coumaric acid, caffeic acid, ferulic acid, quercetin, luteolin, chrysin, galangin, and pinosembrin were found in both honey samples. Furthermore, the detection of higher levels of certain flavonoids (e.g., kaempferol, luteolin, myricetin, and rutin derivatives), particularly in honey sample No. 2, explains the higher total phenolic and flavonoid content of this sample. The literature reports that flavonoids and phenolic acids directly contribute to the total phenolic content of honey and that an increase in the concentration of these compounds shows a strong correlation with total antioxidant capacity [43].

It is known that the biological activity of honey is not dependent on a single component but rather arises from the synergistic effect of phenolic acids, flavonoids, organic acids, enzymes, vitamins, and minerals. Furthermore, the botanical origin of honey, its geographical characteristics, nectar source, and environmental factors play an important role in its phenolic profile and biological activity [42]. It is thought that the differences observed between samples in the study are largely due to these factors.

In conclusion, when the phenolic profile, total phenolic and flavonoid contents, and antioxidant capacity are evaluated together, the honey samples studied are seen to have significant potential, particularly in terms of natural antioxidants and biologically active compounds. These findings support the notion that honey should be considered not only as a nutritional product but also as a functional food and a source of natural biologically active compounds.

One of the main reasons for honey’s inhibitory effect on cholinesterase enzymes is the phenolic acids and flavonoids it contains. It has been reported in the literature that flavonoids such as quercetin, luteolin, kaempferol, pinosembrin, and galangin can interact with the active sites of acetylcholinesterase and butyrylcholinesterase enzymes, thereby exhibiting an inhibitory effect [2,41]. The determination of the presence of these compounds by LC-HRMS analysis in the study supports the inhibition results obtained.

When evaluated in terms of α-glycosidase inhibition, the significant activity observed in honey samples is associated with the potential of caffeic acid, ferulic acid, coumaric acid, and flavonoid derivatives to inhibit enzymes involved in carbohydrate metabolism. It has been reported that these compounds can bind to the active site of enzymes, thereby blocking substrate access and limiting postprandial glucose increases [38].

The literature reports that honey from different geographical origins exhibits moderate inhibitory effects on cholinesterase and α-glycosidase enzymes and that this effect is largely related to phenolic content [42,43]. In this study, the stronger effect of honey sample 2, which has higher phenolic and flavonoid content, on AChE and α-glycosidase inhibition supports this relationship. Furthermore, the similar trend in antioxidant capacity results indicates that phenolic compounds play a role in both antioxidant and enzyme inhibition mechanisms.

The diastase number is an important parameter that provides information about the enzymatic activity and freshness of honey. In the study, the diastase numbers were determined to be 12.2 and 19.6, respectively, which are above the minimum limit of 8. These results indicate that the samples were not subjected to heat treatment and that the enzymatic structure was preserved. Indeed, many studies have reported that the diastase number in natural honey ranges from 10 to 25 [71,72].

The HMF (hydroxymethylfurfural) value is considered an indicator of honey’s storage conditions and whether it has undergone heat treatment. In this study, the HMF value was determined to be 44.728 mg/kg in the first honey sample and 33.765 mg/kg in the second honey sample. According to international standards, an HMF content generally below 40 mg/kg is an accepted criterion for high-quality honey. While the second honey sample was found to be below the codex limits, the first sample was close to the limit value. The literature reports that HMF formation accelerates with storage time and temperature increase. This suggests that the first sample may have been affected by storage conditions or post-harvest processes [73,74]. This situation is particularly associated with storage time and temperature.

Moisture content is a critical parameter for the microbial stability of honey. According to Codex Alimentarius and IHC criteria, the moisture content in honey must be below 20%. Analysis results determined moisture contents of 17% and 15.6%, which are below the maximum 20% limit. These values indicate that the honey is stable against fermentation. The literature reports that the moisture content in mature honey is generally in the range of 14–18% [40]. The values in this study are consistent with the high-quality honey values reported in the literature.

Electrical conductivity values were determined to be 0.221 and 0.176 mS/cm, respectively, and the limit value of 0.8 mS/cm is considered quite normal from a botanical perspective. Electrical conductivity is particularly related to mineral matter and organic acid content, and low values are generally observed in flower honeys [8,44]. The results obtained support this situation.

Free acidity values were found to be 22.14 and 30.11 meq/kg, remaining below the maximum limit of 50 meq/kg. This indicates that the honey is not fermented and that the organic acid balance is within natural limits. pH values were determined to be 3.88 and 3.92. The naturally acidic character of honey is an important factor in inhibiting microbial growth. The literature reports that honey pH values generally range between 3.2 and 4.5 [2,71].

Naphthalene was not detected in either sample in the naphthalene analysis. The absence of naphthalene in honey is important as it indicates that there was no environmental contamination during the production and storage process. According to the food safety approach adopted by the World Health Organization and the FAO, the absence of chemical contaminants in natural products such as honey is a critical criterion for consumer health. Naphthalene is a carcinogenic substance, and the Codex limit for honey is a maximum of 10 µg/kg [12].

When examining the sugar composition, it is seen that fructose and glucose constitute the main sugars in honey. The sum of fructose and glucose was determined to be 71.567% and 75.618%, respectively, and was above the minimum limit of 60%. The very low sucrose and maltose ratios indicate that the honey is mature, natural, and has sufficient invertase activity. The fructose/glucose ratio was determined to be 1.160 and 1.122, respectively, which provides information about the honey’s tendency to crystallize and is consistent with the range of 0.9–1.4 reported in the literature [8].

In a study conducted in 2012, honey samples collected from 14 different geographical regions of South Africa were analyzed for moisture content, pH, proline, and diastase activity. The moisture content of the honeys ranged between 10.09% and 20.73%, pH values ranged from 3.87 to 5.12, proline content varied between 155.7 and 452.4 mg/kg, and diastase activity ranged from 12.72 to 15.01 mL/g/min [70]. When compared with the present study, it was observed that the moisture and pH values are comparable and the diastase activity is consistent, whereas the proline content is relatively lower in the current study.

Overall, the analysis of all physicochemical parameters indicates that the honey samples meet quality, purity, and appropriate storage conditions. The physicochemical data obtained show that the honey samples are largely consistent with the criteria of the International Honey Commission [75] and Codex Alimentarius and also meet the quality and reliability principles recommended by WHO/FAO in terms of food safety. These findings support the conclusion that the honey samples examined are of high quality from both a commercial and biological perspective. International authorities, including Codex Alimentarius, WHO, and FAO, emphasize that honey should be free from veterinary drug residues due to both toxicological risks and their contribution to antimicrobial resistance [76,77]. The International Honey Commission also considers the absence of residues a key quality parameter, highlighting honey as both a nutritional and functional food [8].

Recent studies indicate that antibiotic residues in honey are generally rare or undetectable, largely due to improved beekeeping practices and regulatory control [45,46]. Similar findings have been reported in Türkiye, where most honey samples show no detectable antibiotic contamination [78].

Despite the growing interest in African honeys, most previous studies have focused on individual aspects of honey characterization, such as physicochemical properties, phenolic composition, antioxidant activity, or specific contaminants. Comprehensive investigations integrating physicochemical quality parameters, food safety indicators, antibiotic and naphthalene residues, antioxidant capacity, and enzyme inhibitory activities within the same honey samples remain scarce. In particular, limited information is available on the relationship between the quality and safety characteristics and the biofunctional potential of African honeys. Therefore, a comprehensive assessment addressing these parameters is needed to provide a more complete evaluation of African honey quality, safety, and potential health-promoting properties.

The absence of antibiotic residues in honey is important not only for consumer health, but also because it demonstrates that the product was obtained under natural production conditions and that good beekeeping practices were adopted. The fact that no residues were detected in the honey samples analyzed in this study, despite a broad antibiotic panel, indicates that the samples comply with international quality criteria and are of high quality in terms of food safety. Nevertheless, this work is expected to serve as an important reference for more comprehensive future studies.

A limitation of the present study is the relatively small number of independent honey samples, as only two honey samples were available for analysis. The triplicate measurements performed in the analytical assays represent technical replicates and should not be considered independent biological replicates. Therefore, the findings should be interpreted cautiously and should not be generalized to all honeys from the investigated region. Nevertheless, the comprehensive assessment of physicochemical quality parameters, contaminant residues, antioxidant capacity, enzyme inhibitory activities, and chemical composition provides valuable baseline information on the investigated honey samples.

The limited availability of additional honey samples with well-defined geographical origin, production characteristics, and representative sampling conditions made it difficult to expand the sample set within the scope of the present study. Despite this limitation, the findings provide an important reference for future investigations and may contribute to the development of more comprehensive studies involving larger numbers of independently collected samples. In particular, future research incorporating broader geographical and climatic diversity, different botanical origins, and multiple production periods would be valuable for determining the extent to which the observed chemical and biological characteristics are consistent across honey populations. Such studies, together with increased numbers of independent biological samples and appropriate replication, will enable more robust statistical evaluation and improve the generalizability and representativeness of the findings regarding honey quality, safety, and biofunctional properties.

## 5. Conclusions

The present study comprehensively evaluated two honey samples in terms of physicochemical quality, contaminant status, antioxidant capacity, enzyme inhibition, and metabolite profile. Both samples complied with international standards, confirming good quality, safety, and functional potential. To the best of our knowledge, this study is among the first to comprehensively evaluate the analyzed honey samples by integrating physicochemical quality, antibiotic residues, enzyme inhibitory activity, antioxidant capacity, and metabolomic profiling within a single analytical framework.

Physicochemical parameters (moisture, pH, electrical conductivity, HMF, and diastase activity) were within Codex standards, indicating good quality, proper storage, and minimal heat damage. Sugar profiles confirmed honey maturity and authenticity, with low sucrose levels. No naphthalene residues or detectable veterinary drug contaminants (92 compounds analyzed by LC–MS/MS) were found, confirming product safety.

Both samples exhibited notable antioxidant activity, supported by total phenolic and flavonoid contents, with honey 2 consistently showing stronger effects. Enzyme inhibition assays (AChE, BChE, and α-glycosidase) suggested potential neuroprotective and antidiabetic properties. Metabolomic analysis identified 85 secondary metabolites, including major phenolics such as quercetin, kaempferol, and luteolin, with higher concentrations observed in honey 2, correlating with its enhanced bioactivity.

In summary, the results demonstrate that the analyzed honeys are safe, high-quality, and biologically active, supporting their potential use as functional food products.

## Figures and Tables

**Figure 1:**
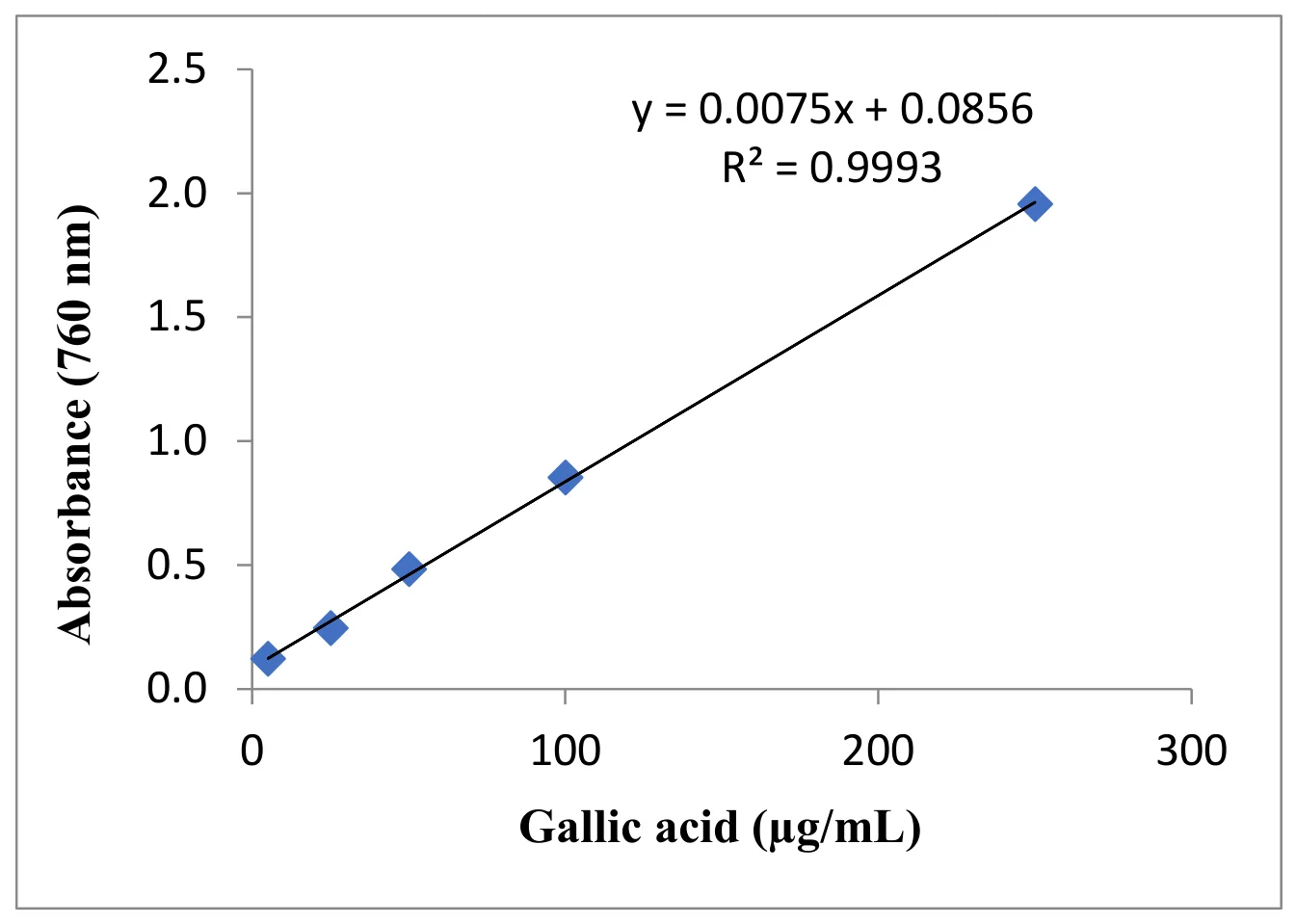
The calibration curve of gallic acid.

**Figure 2:**
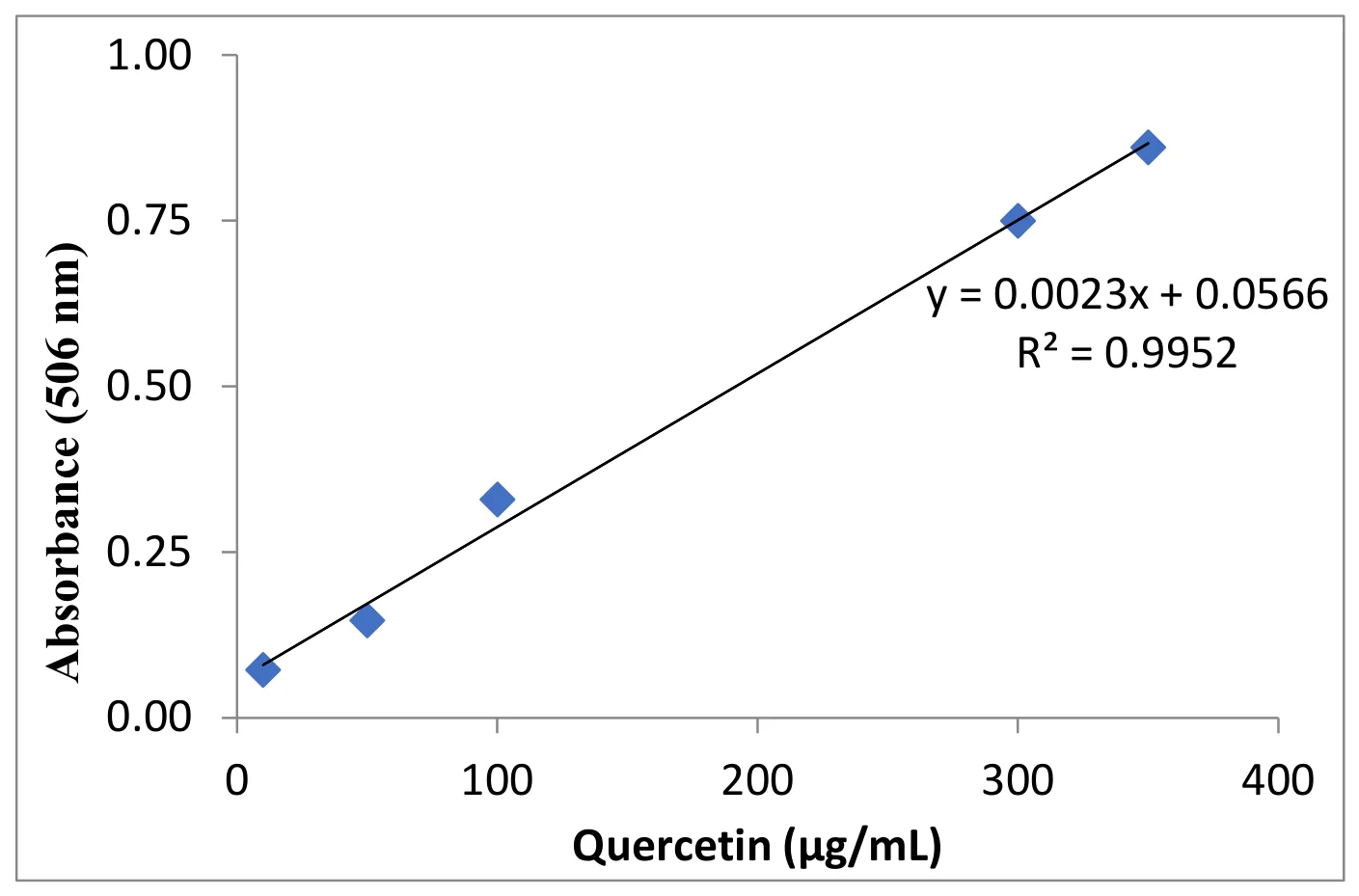
The calibration curve of quercetin.

**Table 1:** Secondary metabolite profile of the honey samples (ng/g).

N.	Secondary Metabolite	R.T. (1)	R.T. (2)	Response (1)	Response (2)	Quan Peak (mz)	1. Honey	2. Honey
1.	Benzoic acid	ND	9.47	ND	57,745,449	121.02950	ND	267.94
2.	4-Hydroxybenzoic acid	7.04	7.04	196,060,769	531,943,683	137.02442	775.14	2190.82
3.	Salicylic acid	9.48	9.48	176,000,892	617,204,689	137.02442	135.38	474.77
4.	Syringic acid	8.41	8.42	51,482,413	71,941,892	197.04555	756.88	1057.67
5.	Vanillic acid	7.93	7.91	50,828,571	50,842,732	167.03498	499.4	499.57
6.	Coumaric acid (trans-3-Hydroxycinnamic acid)	9.21	9.21	225,705,644	448,406,878	163.04007	528.02	1049
7.	Caffeic acid	8.21	8.18	60,587,329	206,862,880	179.03498	101.49	346.5
8.	Caffeic acid phenhyl ester (CAPE)	ND	13.54	ND	9,729,756	283.09758	ND	9.31
9.	Ferulic acid	9.5	9.52	32,195,492	48,406,701	193.05063	168.44	253.25
10.	Sinapic acid	9.6	ND	4,834,110	ND	223.06120	34.22	ND
11.	Chlorogenic acid	7.93	ND	24,703,112	ND	353.08781	242.07	ND
12.	Quinic acid	0.91	ND	153,647,231	ND	191.05611	1513.72	ND
13.	Epigallocatechin gallate	8.21	8.18	52,312,847	207,184,539	179.03508	87.73	347.41
14.	Chrysin (5,7-Dihydroxy-2-phenyl-4H-chromen-4-one)	13.78	13.77	108,293,882	192,647,460	253.05063	100.73	179.73
15.	Apigenin (5,7-Dihydroxy-2-(4-hydroxyphenyl)-4H-chromen-4-one)	12.84	12.84	17,429,869	519,312,999	269.04555	25.67	778.36
16.	Rhoifolin (Apigenin 7-O-neohesperidoside)	ND	11.14	ND	257,755	433.11200	ND	1.2
17.	Rutin hydrate M-OH2	ND	10.62	ND	42,687,783	609.14611	ND	614.52
18.	Luteolin	12.25	12.25	33,130,455	1,211,135,656	285.04046	66.34	2425.16
19.	Diosmetin (Luteolin 4′-methyl ether)	12.99	12.94	6,161,580	42,316,519	299.05611	18.88	130.2
20.	Galangin (3,5,7-Trihydroxy-2-phenyl-4H-chromen-4-one)	13.94	13.93	64,801,881	251,328,820	269.04555	81.21	314.97
21.	Quercetin	11.94	11.95	36,157,071	183,993,841	301.03538	356.56	778.33
22.	İsoquercitrin (Quercetin 3-glucoside)	10.58	ND	894,867	ND	463.08820	9.41	ND
23.	Quercetin 3-rutinoside 7-glucoside	ND	11.15	ND	39,509,664	447.09296	ND	264.06
24.	Isorhamnetin (Quercetin 3′-methyl ether)	12.85	12.84	37,169,572	104,297,521	315.05103	347.42	689.79
25.	Kaempferol	12.68	12.68	64,807,192	5,193,030,889	285.04046	52.04	12,123.28
26.	Afzelin (Kaempferol 3-rhamnoside)	11.75	ND	17,507,152	ND	431.09837	124.29	ND
27.	Kaempferide	14.09	ND	1,692,821	ND	299.05611	2.53	ND
28.	Astragalin (Kaempferol 3-glucoside)	ND	11.15	ND	38,865,013	447.09328	ND	245.09
29.	Myricetin	ND	11.08	ND	27,781,196	317.02982	ND	949.34
30.	Naringenin	11.69	ND	493,126,983	ND	271.06120	763.76	ND
31.	Neohesperidin	ND	10.91	ND	36,698,222	461.07269	ND	805.99
32.	Ellagic acid	ND	11.02	ND	56,502,664	300.99899	ND	411.42
33.	Pinocembrin	13.21	13.2	540,297,062	1,128,479,990	255.06694	277.84	586.12

N.: numbers, R.T.: retention time, ND: not detected. Other analytical data regarding the method are presented in the cited study [27].

**Table 2:** Half-maximal inhibitory concentration (IC_50_) values of honey samples against DPPH^•^ and ABTS**^•+^**.

Samples and Standards	DPPH^•^ Scavenging		ABTS^•+^ Scavenging	
	IC_50_	r^2^	IC_50_	r^2^
BHA	33.521 ± 2.005	0.9751	21.632 ± 1.001	0.9882
BHT	29.742 ± 1.021	0.9893	30.763 ± 2.058	0.9756
α-Tocopherol	27.689 ± 1.012	0.9608	22.542 ± 1.112	0.9962
Trolox	30.152 ± 2.021	0.9852	26.855 ± 2.019	0.9763
1. Honey	41.443 ± 2.185	0.9563	45.1102 ± 3.108	0.9698
2. Honey	32.621 ± 2.045	0.9689	30.335 ± 2.795	0.9589

Data are expressed as mean ± SD of three technical replicate measurements (*n* = 3) for each independent honey sample. These measurements represent technical replicates rather than independent biological samples. Due to the limited number of independent samples, the statistical findings should be interpreted cautiously and should not be generalized beyond the investigated samples. Concentrations are reported in 60 µg/mL.

**Table 3:** Fe^3+^, Cu^2+^ and FRAP reducing power values of honey samples.

Samples and Standards	Fe^3+^		Cu^2+^		Fe^3+^-TPTZ	
	λ_700_	r^2^	λ_450_	r^2^	λ_593_	r^2^
BHA	1.952 ± 0.055	0.9941	1.742 ± 0.025	0.9854	1.850 ± 0.019	0.9856
BHT	1.579 ± 0.081	0.9854	1.455 ± 0.009	0.9723	1.641 ± 0.033	0.9821
α-Tocopherol	2.021 ± 0.055	0.9763	2.101 ± 0.021	0.9863	1.524 ± 0.021	0.9933
Trolox	1.765 ± 0.098	0.9856	1.856 ± 0.018	0.9908	1.385 ± 0.009	0.9765
1. Honey	0.919 ± 0.008	0.9912	1.088 ± 0.011	0.9892	0.856 ± 0.012	0.9544
2. Honey	1.460 ± 0.041	0.9821	1.389 ± 0.015	0.9889	0.997 ± 0.008	0.9872

Data are expressed as mean ± SD of three technical replicate measurements (*n* = 3) for each independent honey sample. These measurements represent technical replicates rather than independent biological samples. Due to the limited number of independent samples, the statistical findings should be interpreted cautiously and should not be generalized beyond the investigated samples. Concentrations are reported in 60 µg/mL.

**Table 4:** Total phenolic and flavonoid contents of honey samples.

Samples	Total Phenolics (µg GAE/g)	Total Flavonoid Contents (µg QE/g)
1. Honey	34.121 ± 1.028	27.854 ± 0.983
2. Honey	57.233 ± 2.022	33.212 ± 0.995

All values are expressed as mean ± SD of three technical replicate measurements (*n* = 3) for each independent honey sample. GAE: gallic acid equivalents; QE: quercetin equivalents.

**Table 5:** Inhibitory effects of honey samples on AChE, BChE and α-glycosidase enzymes.

Samples and Standards	AChE		BChE		α-Glycosidase	
	IC_50_	r^2^	IC_50_	r^2^	IC_50_	r^2^
1. Honey	11.33	0.9879	13.11	0.9859	10.71	0.9812
2. Honey	8.21	0.9956	13.63	0.9895	9.33	0.9970
Tacrine	3.12	0.9752	4.32	0.9921	-	-
Acarbose	-	-	-	-	4.21	0.9952

Concentrations are given in μg/mL. Tacrine was used as a standard inhibitor for AChE and BChE, and acarbose for α-glycosidase.

**Table 6:** Proline, diastase, HMF, moisture, electrical conductivity, free acidity, pH, naphthalene and sugar results of honey.

Analyzes	1. Honey	2. Honey
Proline (mg/kg)	521.854 ± 15.245	759.95 ± 21.198
Diastase	12.2 ± 0.385	19.6 ± 0.339
HMF (mg/kg)	44.728 ± 2.142	33.765 ± 1.983
Moisture (%)	17 ± 0.24	15.6 ± 0.56
Electrical conductivity (mS/cm)	0.221 ± 0.012	0.176 ± 0.008
Free acidity (meq/kg)	22.14 ± 1.12	30.11 ± 1.51
pH	3.88	3.92
Naphthalene (µg/kg)	N.D.	N.D.
Glucose (%)	33.125 ± 0.334	35.624 ± 0.298
Fructose (%)	38.442 ± 0.442	39.994 ± 0.451
Sucrose (%)	0.104 ± 0.001	0.132 ± 0.002
Maltose (%)	1.103 ± 0.072	1.084 ± 0.064
Fructose + Glucose (%)	71.567	75.618
Fructose/Glucose	1.160	1.122

N.D.: Not determined. TS: Turkish standard. The ± values represent standard deviations obtained from technical replicates.

**Table 7:** Drug residues in the honey samples (µg/kg).

No	Name	Result	LOQ	No	Name	Result	LOQ
1	4-Epi CTC	ND	1.56	47	Sulfadimethoxine	ND	1.56
2	4-Epi OTC	ND	1.56	48	Sulfadoxine	ND	1.56
3	4-Epi TC	ND	1.56	49	Sulfamerazine	ND	1.56
4	Albendazol	ND	1.56	50	Sulfamethazine	ND	1.56
5	Amoxicillin	ND	1.56	51	Sulfamether	ND	1.56
6	Amprolium	ND	1.56	52	Sulfamethizole	ND	1.56
7	Anthyroerythromycin	ND	1.56	53	Sulfamethoxazole	ND	1.56
8	Carbadox	ND	1.56	54	Sulfamethoxypyridazine	ND	1.56
9	Chloramphenicol	ND	1.56	55	Sulfamonomethoxin	ND	1.56
10	Chlortetracycline	ND	1.56	56	Sulfamoxol	ND	1.56
11	Ciprofloxacin	ND	1.56	57	Sulfaphenazole	ND	1.56
12	Danofloxacin Mesylate	ND	1.56	58	Sulfapyridine	ND	1.56
13	Dapson	ND	1.56	59	Sulfaquinoxalin	ND	1.56
14	Diclazuril	ND	1.56	60	Sulfatcetamide	ND	1.56
15	Dicloxacillin	ND	3.125	61	Sulfathiazole	ND	1.56
16	Difloxacin HCl	ND	1.56	62	Sulfichloropyridazine	ND	1.56
17	Dihyrostreptomycin	ND	1.56	63	Sulfisomidine	ND	1.56
18	Doksisiklin	ND	1.56	64	Sulfisoxazole	ND	1.56
19	Enrofloxacin	ND	1.56	65	Tetrasiklin	ND	1.56
20	Eritromisin	ND	1.56	66	Thiamphenicol	ND	1.56
21	Fenbendazole	ND	1.56	67	tiamulin	ND	1.56
22	Florfenicol	ND	1.56	68	Tilmicosin	ND	1.56
23	Flumequine	ND	1.56	69	Tolfenamic	ND	1.56
24	Ipronidazole	ND	1.56	70	Toltrazuril	ND	1.56
25	Levasimol	ND	1.56	71	Triclabendazol	ND	1.56
26	Maduramisin	ND	1.56	72	Trimethoprim	ND	1.56
27	Marbofloxacin	ND	1.56	73	Tylasmphophate	ND	1.56
28	Metronidazole	ND	1.56	74	Tylosin	ND	1.56
29	Monensin	ND	1.56	75	Metronıdazol-Oh	ND	1.56
30	Nicarbazin	ND	1.56	76	Carprofen	ND	1.56
31	Oksitetrasiklin	ND	1.56	77	Clındamycın	ND	1.56
32	Olaquindox	ND	1.56	78	Lıncomycın	ND	1.56
33	Ormetoprim	ND	1.56	79	Sulfachloropyrıdazıne	ND	1.56
34	oxfendazol	ND	1.56	80	Nalıdıxıc Acıd	ND	1.56
35	Oxolinic acid	ND	1.56	81	3-Methyl-Quınnoxacalıne-2-Car. Acıd	ND	1.56
36	Phenyl butazone	ND	1.56	82	Spectınomycın Sulphate Tetrahydrate	ND	1.56
37	Ronidazole	ND	1.56	83	Gentamycın Sulfate	ND	1.56
38	Salinomisin	ND	1.56	84	Ampıcıllın Trıhydrate	ND	1.56
39	Sarafloxacin HCl	ND	1.56	85	Metamızol Sodyum Monohydrate	ND	1.56
40	Sipiramisin	ND	1.56	86	Oleandomycın	ND	1.56
41	Streptomycin	ND	1.56	87	Tylocın B	ND	1.56
42	Succinlysulfathiazole	ND	1.56	88	Lomefloxacın	ND	1.56
43	Sulfachlorpyrida	ND	1.56	89	Meloxıcam	ND	1.56
44	Sulfachmoxalin	ND	1.56	90	Neomycın Sulphate	ND	1.56
45	Sulfaclozine	ND	1.56	91	Ampıcıllın Trıhydrate	ND	1.56
46	Sulfadiazine	ND	1.56	92	Penıcıllın V	ND	1.56

ND: Not determined. LOQ = limit of quantification. The analytical parameters were previously established and validated in the study conducted by İzol and were adopted in the present study [27].

## Data Availability

The original contributions presented in this study are included in the article.
